# Unbounding the mental number line—new evidence on children's spatial representation of numbers

**DOI:** 10.3389/fpsyg.2013.01021

**Published:** 2014-01-22

**Authors:** Tanja Link, Stefan Huber, Hans-Christoph Nuerk, Korbinian Moeller

**Affiliations:** ^1^Department of Psychology, Eberhard Karls UniversityTuebingen, Germany; ^2^Knowledge Media Research CenterTuebingen, Germany

**Keywords:** mental number line, number line estimation, estimation strategies, proportion judgment, numerical development

## Abstract

Number line estimation (i.e., indicating the position of a given number on a physical line) is a standard assessment of children's spatial representation of number magnitude. Importantly, there is an ongoing debate on the question in how far the bounded task version with start and endpoint given (e.g., 0 and 100) might induce specific estimation strategies and thus may not allow for unbiased inferences on the underlying representation. Recently, a new unbounded version of the task was suggested with only the start point and a unit fixed (e.g., the distance from 0 to 1). In adults this task provided a less biased index of the spatial representation of number magnitude. Yet, so far there are no children data available for the unbounded number line estimation task. Therefore, we conducted a cross-sectional study on primary school children performing both, the bounded and the unbounded version of the task. We observed clear evidence for systematic strategic influences (i.e., the consideration of reference points) in the bounded number line estimation task for children older than grade two whereas there were no such indications for the unbounded version for any one of the age groups. In summary, the current data corroborate the unbounded number line estimation task to be a valuable tool for assessing children's spatial representation of number magnitude in a systematic and unbiased manner. Yet, similar results for the bounded and the unbounded version of the task for first- and second-graders may indicate that both versions of the task might assess the same underlying representation for relatively younger children—at least in number ranges familiar to the children assessed. This is of particular importance for inferences about the nature and development of children's magnitude representation.

## Introduction

The metaphor of a mental number line (Moyer and Landauer, 1967; Restle, 1970) describing the (spatial) representation of number magnitude is widely recognized (for overviews see Hubbard et al., 2005; De Hevia et al., 2006) and also considered in the currently most influential model in numerical cognition research [i.e., the Triple Code Model (Dehaene, 1992; Dehaene and Cohen, 1997; Dehaene et al., 2003)]. Behavioral (e.g., Dehaene et al., 1993; Fischer, 2001, 2003) as well as neuropsychological (e.g., Zorzi et al., 2002) data provide evidence for an automatic activation of number magnitude on an analogous left-to-right oriented number line in Western cultures (see Shaki et al., 2009, for other cultures). Against this background, it is interesting to take a closer look at the development of the mental number line representation in children.

A standard task to make inferences about the development of the mental number line is the number line estimation task (e.g., Siegler and Opfer, 2003; Geary et al., 2008; Whyte and Bull, 2008; see also Petitto, 1990) also known as number-to-position task (e.g., Berteletti et al., 2010). In this task participants are required to estimate the spatial position of a given number on an empty number line with labeled endpoints defining the numerical range covered (e.g., 0–100; e.g., Siegler and Opfer, 2003). Usually, the deviance of the estimated position of a number from its correct position is interpreted to provide information about how the mapping of numbers to space and thus the mental number line representation develops. Generally, estimation performance is more error-prone in younger children as they tend to overestimate the position of relatively small numbers to the right (i.e., placing 9 at about the position of 40; Moeller et al., 2009). As a consequence, the positions of relatively large numbers are compressed toward the end of the scale which results in relatively high estimation errors (Siegler and Opfer, 2003; Booth and Siegler, 2006; Laski and Siegler, 2007). To account for this estimation pattern, Siegler and colleagues proposed children's estimations to represent a quite isomorphic reflection of a logarithmic underlying representation of number magnitude as the authors found a logarithmic function to fit the observed estimation pattern best. With increasing age and experience, however, the authors suppose children to develop a linear representation of number magnitude reflected by an estimation pattern fitted best by a linear function. This representational change, also referred to as log-to-linear shift, is interpreted to reflect the development toward a linear representation of number magnitude in older children and adults (Siegler and Opfer, 2003; Siegler and Booth, 2004; Booth and Siegler, 2006, 2008).

However, the conclusion of such a representational shift as drawn by Siegler and colleagues is currently discussed controversially with respect to both theoretical but also methodological issues (e.g., Barth and Paladino, 2011; Barth et al., 2011; Moeller and Nuerk, 2011; Slusser et al., 2013; see also Ebersbach et al., 2013 for an overview). From a theoretical point of view, there are alternative accounts to explain the developmental changes in number line performance. A seemingly logarithmic response pattern may be accounted for by a two- or multi-linear fitting procedure, while a seemingly linear pattern may be accounted for by proportion judgment. As regards logarithmic fitting, Moeller et al. (2009; see also Helmreich et al., 2011) observed that a two-linear model suggesting separate but linear representations for one- and two-digit numbers predicts the estimation performance of first-graders in a 0–100 number line task even better than a logarithmic model. Theoretically speaking, the results of Moeller and colleagues do not indicate children's estimation pattern to directly reflect their spatial representation of number magnitude. Rather, they emphasize the importance of understanding the place-value structure of the Arabic number system: With increasing age and experience children master the integration of tens and units into the base-10 place-value structure of the Arabic number system and the separate representations are then integrated to result in a linear estimation pattern (Moeller et al., 2009; Helmreich et al., 2011; see also Ebersbach et al., 2008, for a similar two-linear approach). Another argument challenging the hypothesis of a representational log-to-linear shift was suggested by Barth and Paladino (2011) addressing seemingly linear fittings (see also Slusser et al., 2013). These authors suggested the standard number line estimation task to be more of a proportion judgment than a number magnitude estimation task. Barth and her colleagues argue that the to-be-estimated numbers are not considered in isolation but always in relation to reference points such as the start and endpoint of the given number line or its half. Their claim is methodologically corroborated by fitting results for power models usually used in proportion-judgment context (e.g., Spence, 1990) which provided the best fit for children's estimation performance: In contrast to a linear model which cannot account for systematic biases at reference points the authors found that either one- (considering start and endpoint as references; cf. Spence, 1990) or two-cycle power models (i.e., considering start and endpoint as well as their mean as reference points; cf. Hollands and Dyre, 2000) fitted 7-year-old children's estimation patterns on a 0–100 scale best (Barth and Paladino, 2011). From a theoretical point of view, Barth and colleagues suppose the standard (bounded) number line estimation task to reflect the application of proportion-judgment strategies rather than providing a direct measure of the spatial representation of number magnitude. This is corroborated by the finding that with increasing age more reference points are considered for estimation performance (Slusser et al., 2013).

The argument that the traditional number line estimation task induces strategies of proportion judgment was further corroborated by Cohen and Blanc-Goldhammer (2011). They observed smaller standard deviations of adults' estimations close to reference points but larger standard deviations between these points resulting in a characteristic *M*-shaped distribution. The validity of such a pattern to indicate the use of reference points was also corroborated when evaluating eye fixation data (see Schneider et al., 2008, for children's eye fixation data; see also Sullivan et al., 2011, for adult data).

Considering a recently introduced new version of the number line estimation task (see below for more details) one aim of the current study was to evaluate whether proportion-judgment strategies found in bounded number line estimation are a generalizable characteristic of number line estimation and how the application of this strategy is related to age.

Despite the debate on the nature of the numerical representations and processes underlying number line estimation performance there is accumulating evidence suggesting that number line estimation performance is not only systematically related to actual numerical performance but also predictive of future numerical development. For instance, the acuity of children's mental number line representation as assessed by the linearity of children's number line estimations was found to be positively correlated with other numerical competencies such as numerosity estimation or numerical magnitude comparison (Booth and Siegler, 2006; Laski and Siegler, 2007) but also more complex arithmetic indices such as actual addition performance (Booth and Siegler, 2008). In the same study, children's number line estimation performance was also a reliable predictor of the ability to learn new addition problems (Booth and Siegler, 2008; see also Gunderson et al., 2012; Muldoon et al., 2013, for longitudinal evaluations of the relationship between number line estimation and children's mathematical development). Finally, there is now even first evidence from intervention studies suggesting a causal relationship between the acuity of the mental number line representation and more complex numerical/arithmetic abilities. For instance, Siegler and Ramani (2009; see also Ramani and Siegler, 2011) observed that playing simple linear number board games not only improved children's number line estimation performance significantly but also that this training effect generalized to their arithmetic competency (see also Fischer et al., 2011, for the validity of embodied experiences of spatial number magnitude; Kucian et al., 2011, for similar evidence in children with dyscalculia).

Taken together, it can be noted that number line estimation performance is a reliable predictor of actual and future numerical competencies even though it is still under debate what exactly is assessed by the number line estimation task in its standard bounded version with given start- and endpoint.

Cohen and Blanc-Goldhammer (2011; see also Booth and Siegler, 2006 for a somewhat similar task) proposed a new unbounded version of the number line estimation task without a predefined fixed endpoint. Instead, a unit (i.e., the distance between 0 and 1) is given together with a start point allowing for the estimation of the spatial position of a presented target number on a number line. Importantly, evaluation of participants' estimation pattern corroborated their hypothesis that this task version provided a less biased measure of the mental number line representation: There were no indications of systematic biases reflecting the use of reference points. Moreover, variability of participants' estimation errors increased continuously with number magnitude. This is in line with the assumption of a linear mental number line representation with scalar variance (Gibbon, 1977; Gibbon and Church, 1981; Whalen et al., 1999). This scalar variance hypothesis suggests that the spacing between adjacent numbers on the mental number line is equidistant while representational uncertainty increases with the magnitude of the numbers. Against this background, the authors concluded that the unbounded number line estimation task seems to provide a more pure measure of the underlying mental number line representation as compared to the traditional bounded version of the task.

The only published data on the unbounded number line estimation task are from adult participants, however, number line estimation tasks are used much more prominently in the assessment of children's mental number line representation. Therefore, the objectives of the current study were straightforward.

We wished to evaluate how far the results of Cohen and Blanc-Goldhammer (2011) generalize to children's estimation performance. Therefore, we recruited a broad sample of primary school children from grade one through four as well as a sample of adult controls to perform both tasks, the new unbounded and the standard bounded version of the number line estimation task. Because there are no data available on children performing the unbounded number line estimation task, hypotheses were derived from recent data for the bounded number line estimation task. Slusser et al. (2013) observed children at the age of 7–8 to make use of 2 or 3 reference points (start-, end- and midpoint) to increase their estimation accuracy. In contrast, for younger children's estimation patterns indications for such a proportion-judgment strategy were less obvious. For five-year-olds the authors even suggested that children might have ignored the endpoint of the scale treating the task as an ‘open-ended magnitude judgment’ (Slusser et al., 2013, p. 203) comparable to an unbounded version of the number line estimation task. Furthermore, in an eye-tracking study Schneider et al. (2008) corroborated the assumption of qualitative differences between estimation strategies between relatively younger and older children. In particular, they observed that third-graders targeted their eye fixations more directly toward to-be-expected reference points (i.e., start, middle, and endpoint of the number line) than did younger children. Against this background, we expected a change in estimation strategies in the bounded number line task to occur from grade three at the latest with more pronounced indications for the use of reference points for older children. In contrast, based on the results for the unbounded number line estimation task in adults (Cohen and Blanc-Goldhammer, 2011) indicating a less biased measure of number line estimation no such qualitative change of estimation strategy was expected for the unbounded number line estimation task.

To pursue these hypotheses we evaluated two different aspects of our participants' estimation performance in accordance with the proceeding of Cohen and Blanc-Goldhammer (2011). First, we appraised participants' estimation patterns by fitting different kinds of models. Additionally, we considered the distribution of participants' estimation errors. Taken together, these two aspects should answer the questions whether there are qualitative differences in solution strategies (number line estimation vs. proportion judgment) between (i) the bounded and the unbounded version of the number line estimation task in children and (ii) at what age such differences emerge.

## Methods

### Participants

A cross-sectional sample of 233 primary school children [65 first-graders (31 girls; mean age: 6;7 years, *SD* = 3.90 months), 61 second-graders (32 girls; mean age: 7;7 years, *SD* = 5.58 months), 59 third-graders (23 girls; mean age: 8;8 years, *SD* = 6.35 months) and 48 fourth-graders (27 girls; 9;8 years, *SD* = 5.91 months)] was assessed on a battery of basic numerical tasks including number line estimation to investigate the development of numerical competencies. Children were tested three months after class started. All children participated voluntarily and were included in the sample only after their parents provided a signed informed consent form. In addition, a control sample of 68 university students (56 females; mean age: 23;5 years, *SD* = 4;8 years) volunteered to perform the number line tasks.

### Stimuli

**Figure 1:**
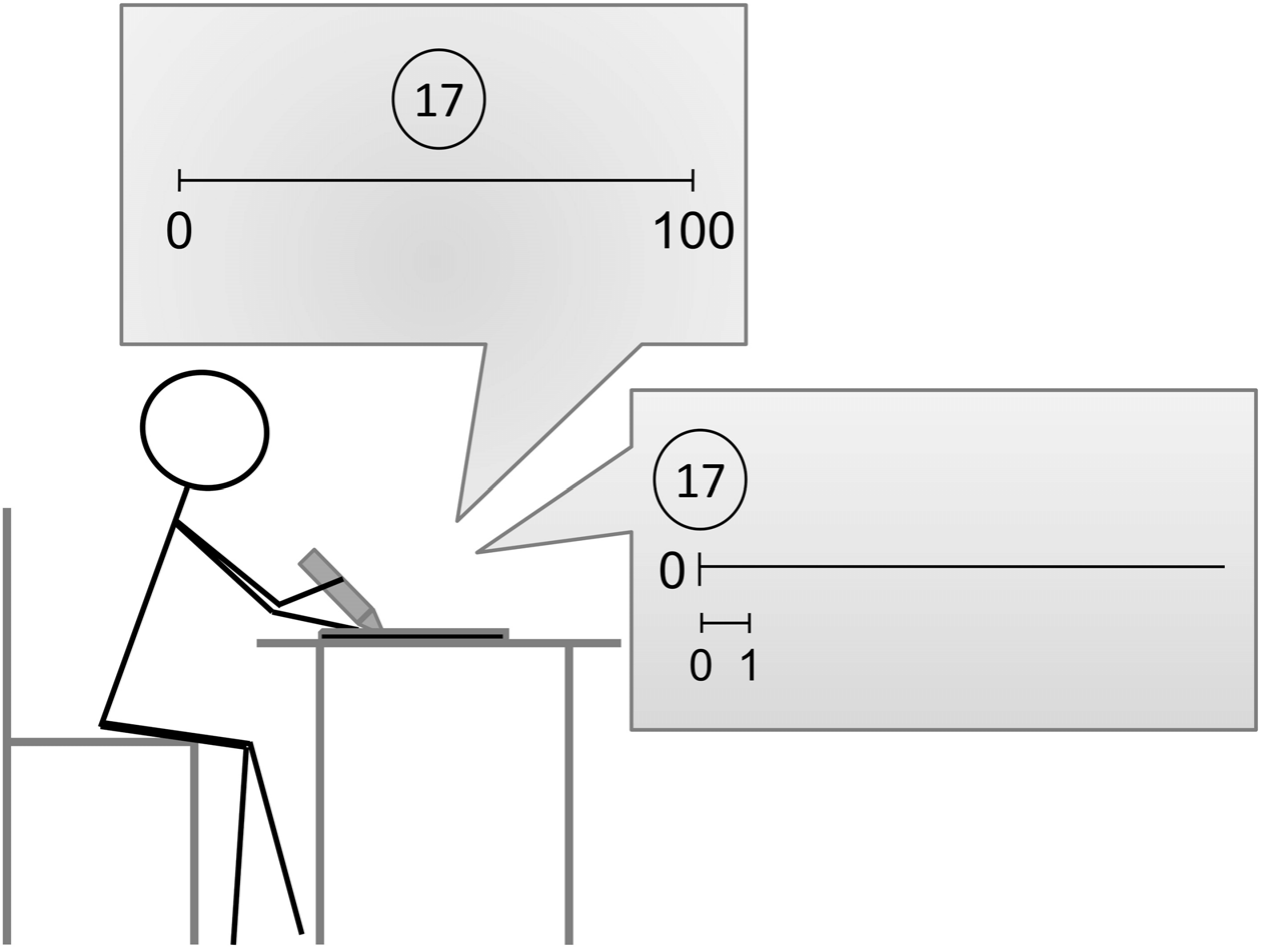
**Schematic illustration of the bounded number line task and the new unbounded version of the task**.

For the different age groups we used different number scales covering the ranges that are taught in the respective grades and can thus be considered more or less familiar to the children tested, this means that children should possibly be able to infer the midpoint of the respective range (first-graders: 0–10; second-graders: 0–20; third-graders: 0–100; fourth-graders: 0–1000; adults: 0–10,000). At all age groups 20 stimuli for the bounded number line task were chosen to allow for reliable identification of possible proportion-judgment strategies, resulting in more items at the suggested reference points (cf. Barth et al., 2011). A total of four items was displayed on one DIN A4 sheet with the start-point of the number lines being varied horizontally to prevent participants from relying on estimates of previous trials as possible anchor points. All number lines were 20 cm long with labeled endpoints below and the to-be-estimated number placed above the middle of the number line (see Figure 1 for a schematic illustration). In all number ranges two practice items (exception: number range 0–10 with only one practice item) ensured participants understanding of the task and were shown on the first page prior to the critical trials. Different from the bounded number line estimation task we did not change the range covered by the unbounded number line task for the different age groups. The same physical length of 20 cm was used for the unbounded and the bounded task to enhance comparability between task versions. The unit indicating the distance between 0 and 1 was depicted below the start-point. The to-be-estimated numbers were presented above the start point (see Figure 1 for a schematic illustration). The numerical length of the unbounded number line was 29. Only items in the range from 0 to 20 were used to assess unbounded number line performance, leaving enough space between the largest numbers and the physical endpoint. A total of 15 items were presented as numbers 0, 1, 5, 11, and 20 were excluded and 10 served as practice item. Again, four items were presented on one DIN A4 sheet arranged in the same way as the bounded task items.

### Procedure

All tasks were administered in group settings. For the bounded number line task, children received the oral instruction that they are presented with a special number line which only has a start- and endpoint but no further numbers in between. Then the task was explained (in German) as follows: “Look at the number printed above the number line—where do you think this number goes between 0 and X. Please mark your estimate on the line.” Importantly, no numbers apart from the start and the endpoint were indicated. In the unbounded task, the instruction was similar: Children were told that there is no end to the number line but that they can see how long the distance from 0 to 1 is. Further instructions were adapted to the task: “Look at the number printed above the number line—where do you think this number goes?” Again, no other numbers were indicated to participants. Adults were provided with written instructions. Neither age group received feedback as to the correctness of any of the items. Task order was the same for all age groups: participants started with the bounded followed by the unbounded number line estimation task.

### Analyses

As variables of interest we evaluated children's mean estimates (indicating their estimation performance) as well as the standard deviation of children's percent absolute error (PAE = |Estimate − Target number|/ Scale ^*^100; cf. Siegler and Booth, 2004, reflecting the variability of their estimates). In line with the procedure of Ashcraft and Moore (2012) we ran a contour analysis contrasting the variability of children's estimation errors at and in between possible reference points (using *t*-tests) for both the bounded and unbounded estimation task separated for each age group. Therefore, standard deviations of the PAEs of the two target numbers closest to the origin, the first quartile, the midpoint, the third quartile and the endpoint, were pooled. In case target number and reference point were identical (e.g., item 15 in the unbounded task conforms to the third quartile) the item itself plus the two closest target numbers were considered. For first-graders' on the 0–10 bounded number line task only one item was considered for the origin and endpoint, respectively, as there were only 8 target numbers.

In addition, we evaluated the goodness of fit of several models used in previous studies to mathematically reflect children's estimation performance (e.g., linear, power models, etc.). To fit models Matlab 7.14 was used applying the trust region algorithm for the fitting of non-linear models. For both number line tasks we estimated the fit of the same models thereby differentiating grossly between three families of functions corresponding to different estimation strategies: (i) direct estimation strategies should be indicated by the superior fit of the linear and (unbounded) power function (cf., Slusser et al., 2013), (ii) proportion-judgment based estimation strategies should be indexed by one- and two-cycle power models (cf. Barth et al., 2011), and (iii) dead-reckoning strategies should be reflected by dual and multi scallop models (for scallop models see Cohen and Blanc-Goldhammer, 2011).

Linear models were fitted with two free parameters (i.e., the intercept and the slope). The unbounded power model had one free parameter (i.e., the exponent) while dual scallop and multi scallop models were fitted with two free parameters (i.e., the exponent and the size of the working window). The linear and the unbounded model allow for identifying direct estimation strategies, with no application of an additional strategy like dead-reckoning or proportion judgment. In contrast, cyclic power models suggest that participants use at least two references point (start and end point for the one-cycle model whereas the two-cycle model indicates the use of an additional central reference point). Cyclic models were fitted with one free parameter (i.e., the exponent determining the shape of the power function). Dual and multi scallop models are well suited to find out whether participants applied a dead-reckoning strategy. Thereby, participants first estimate a particular working window of numbers (e.g., 5) and then use multiplies of this working window to estimate the position of higher numbers. The dual scallop model allows for identifying participants, who applied the working window twice and the multi scallop model participants, who repeated their working window multiple times. As identification of such dead-reckoning strategies was not at the heart of this study, we summarized results of the respective models in the category of “others.”

However, different from testing the scallop models in the bounded condition applying cyclic power models in the unbounded task is not as straightforward as it seems to be at first glance. Importantly, cyclic power models require definition of an upper bound, which can be easily specified in a bounded number line task. However, for the unbounded number line task such an upper bound does not exist *per se*. Theoretically, participants might have used the end of the physical line as an upper bound or the largest number which they had to estimate. Since these strategies might vary between participants, a fixed upper bound for testing cyclic models in the unbounded task cannot be used. Therefore, this upper bound has to be estimated by the fitting procedure (cf. Barth et al., 2011). The range of the parameter accounting for the upper bound was allowed to vary between 19 and 29, corresponding to the largest target number (19) and the numerical end of the line (29).

Models were compared by calculating AICc (Akaike information criterion with a correction for finite sample sizes) values for each participant (e.g., Burnham et al., 2011; see also Cohen and Blanc-Goldhammer, 2011, for a similar procedure). Lower AICc values were then interpreted as superior fit of either model^1^.

## Results

In total, 61 participants (18 first-graders, 18 second-graders, five third-graders, five fourth-graders and 15 adults) were excluded from final analyses as they had missing data on at least three items within one of the tasks and/or showed an estimation pattern indicating insufficient understanding of the task (e.g., marking the middle of the number line for all trials). Furthermore, individual estimates that differed more than ± 3 SD from the age groups' mean estimate were also excluded. It is important to note that this trimming procedure did not change results substantially.

### Estimation patterns and model fittings

**Figure 2:**
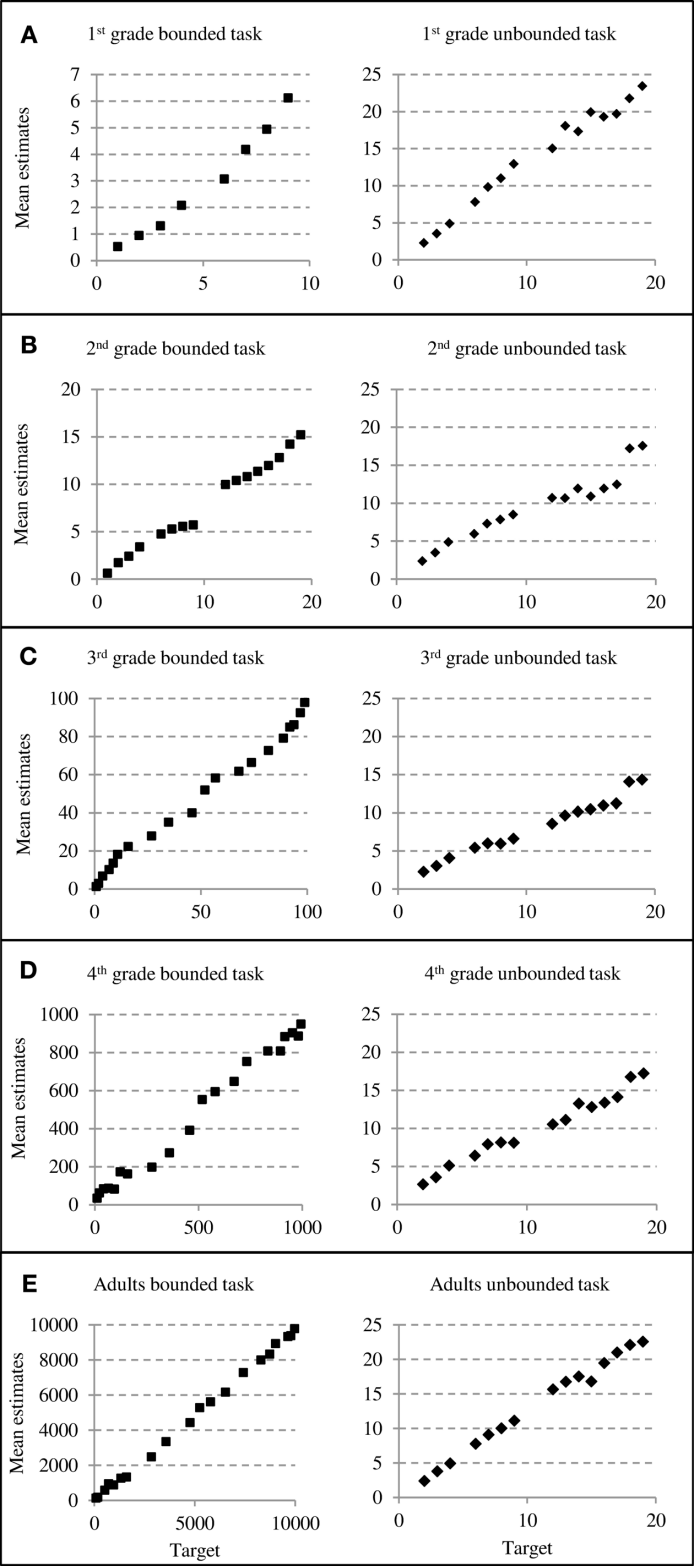
**Estimation patterns for both versions of the number line estimation task**. The left column depicts bounded and the right column depicts unbounded number line estimates for all age groups **(**grade one through adults: **A**–**E)**.

Mean estimates were calculated separately for all age groups and plotted as a function of target number to look for obvious indications of the use of reference points (see Figure 2). We found that for all age groups mean estimates increased steadily with increasing size of the target number independently of task version. Only first graders' bounded number line estimates obviously differed in distribution from older children's and adults' bounded estimates (see Figure 2A, left column). In general, first graders seemed to underestimate larger numbers as they did not produce estimates larger than about 6 on the 0–10 bounded number scale.

For the unbounded number line task the distributions of estimates look very similar for the different age groups (see Figures 2A–E, right column). It is notable, that first-graders and adults overestimated numbers toward the end of the scale (i.e., numbers close to 20; see Figure 2A, right column), seemingly they often tended to locate larger numbers toward the end of the physical line (which was at 29).

Because plotting estimates as a function of target number only allows for visual inspection to conclude whether children did or did not use proportion-based estimation strategies, we fitted estimates with different types of models. Table 1 depicts frequencies of best fitting models for the different age groups separated for both, task and strategy applied (parentheses show relative frequencies).

**Table 1:** **Absolute and relative frequency (percentages) of best fitting models indicating direct estimation, proportion judgments or other estimation strategies (left column) and detailed distribution of best fitting model of participants' estimates (right column) separated for bounded and unbounded number line tasks and age groups**.

	**Estimation strategies**			**Model fittings**					
	**Direct**	**Proportional**	**other**	**Linear**	**Unbounded power**	**Dual scallop**	**Multi scallop**	**One- cycle**	**Two- cycle**
**BOUNDED TASK**									
1st grade	**40 (85)**	7 (15)	0	**30 (64)**	10 (21)	0	0	4 (9)	3 (6)
2nd grade	**32 (74)**	9 (21)	2 (5)	**22 (51)**	10 (23)	1 (2)	1 (2)	5 (12)	4 (9)
3rd grade	17 (31)	**37 (69)**	0	4 (7)	13 (24)	0	0	**25 (46)**	12 (22)
4th grade	15 (35)	**28 (65)**	0	5 (12)	10 (23)	0	0	8 (19)	**20 (47)**
Adults	**37 (70)**	16 (30)	0	12 (23)	**25 (47)**	0	0	7 (13)	9 (17)
**UNBOUNDED TASK**									
1st grade	**40 (85)**	4 (9)	3 (6)	15 (32)	**25 (53)**	2 (4)	1 (2)	4 (9)	0
2nd grade	**39 (91)**	4 (9)	0	17 (40)	**22 (51)**	0	0	3 (7)	1 (2)
3rd grade	**50 (93)**	0	4 (7)	12 (22)	**38 (70)**	2 (4)	2 (4)	0	0
4th grade	**39 (91)**	2 (5)	2 (5)	10 (23)	**29 (67)**	1 (2)	1 (2)	2 (5)	0
Adults	**45 (85)**	2 (4)	6 (11)	16 (30)	**29 (55)**	2 (4)	4 (8)	2 (4)	0

*The best fitting models are indicated in bold script*.

First- and second graders seemed to use a direct estimation strategy solving the bounded number line estimation task as the linear model provided the best fit (for 64% of the first- and 51% of the second-graders). Interestingly, this pattern changed for third- and fourth-graders: Only 31% of the third-graders' and 35% of the fourth-graders' estimates were accounted for best by models indicating direct estimation strategies (i.e., linear and unbounded power models). Instead, one- or two-cycle models provided a better fit, clearly indicating the use of reference points and thus proportion-judgment strategies (Barth and Paladino, 2011). In detail, 68% of third-graders' estimates were fitted best by cyclic power models: 46% by one-cycle and 22% by two-cycle models. Thus, most third-graders seemed to consider two reference points (i.e., start- and endpoint). Moreover, 66% of fourth-graders' estimates were also accounted for best by cyclic power models. The high percentage for two-cycle models (47%) indicated the prominent use of three reference points.

Unexpectedly, a direct estimation strategy was also observed for the majority of adult's estimates (70%) as the single scallop model provided the best fit for 47% of participants' estimates. At first glance, this result pattern seems to contradict our hypothesis and also previous results of Cohen and Blanc-Goldhammer (2011). However, a closer look at the estimation pattern clarified this: as adults show estimation patterns with very small PAEs all model fittings were more or less identical as indicated by the respective adjusted *R*^2^ (linear model: *mean adj.R*^2^ = 0.985, unbounded power model: *mean adj.R*^2^ = 0.984, dual scallop model: *mean adj.R*^2^ = 0.983, multi scallop model: *mean adj.R*^2^ = 0.983, one-cycle model: *mean adj.R*^2^ = 0.981, two-cycle model: *mean adj.R*^2^ = 0.981). Thus, as power models with an exponent of 1 are basically similar to a linear function without an intercept, selection of best fitting model does not provide sufficient evidence to reliably differentiate between estimation strategies. Yet, a closer inspection of (adults') estimation errors is informative (see below).

Regarding model fittings for estimates in the unbounded version, results are more consistent: Independent of age group, the majority of participants was always classified to use direct estimation strategies as an unbounded power model provided the best fit for their estimates. According to Cohen and Blanc-Goldhammer (2011), this model indicates that participants directly estimated targets' locations (see also Slusser et al., 2013). Dual and multi scallop models fitted best for only a few participants' estimates, most frequently adults, as did cyclic models. Taken together, these data do not corroborate the notion of a prominent use of specific strategies such as proportion-judgment or dead-reckoning in unbounded number line estimation.

### PAE distribution

**Figure 3:**
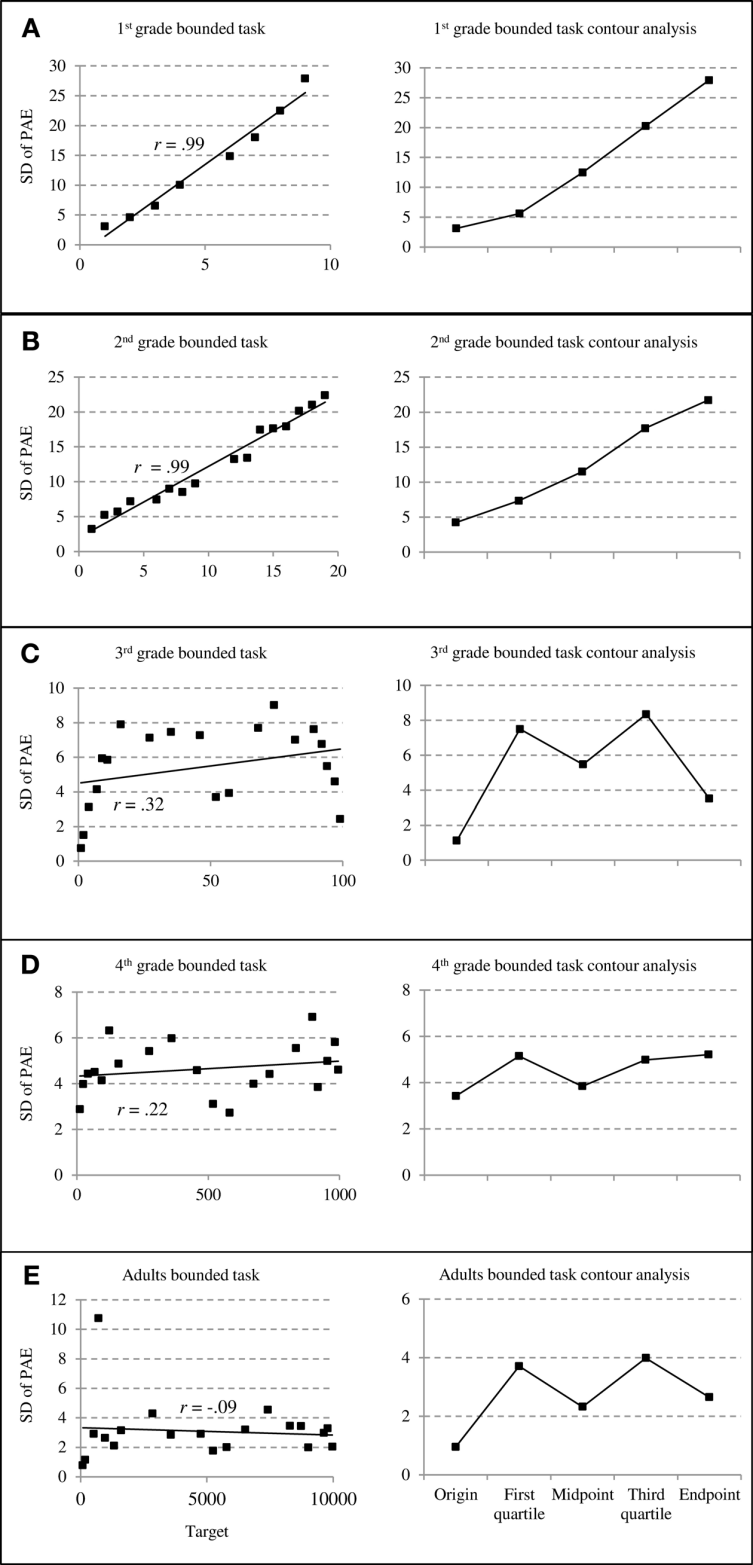
**Standard deviations of percent absolute errors (PAE) for bounded number line estimation performance**. The left column depicts the relationship between standard deviations of PAEs and target numbers whereas the right column depicts results of counter analyses summarizing standard deviations of PAEs at specific reference points **(**cf. Ashcraft and Moore, 2012; grade 1 through adults: **A**–**E)**.

**Figure 4:**
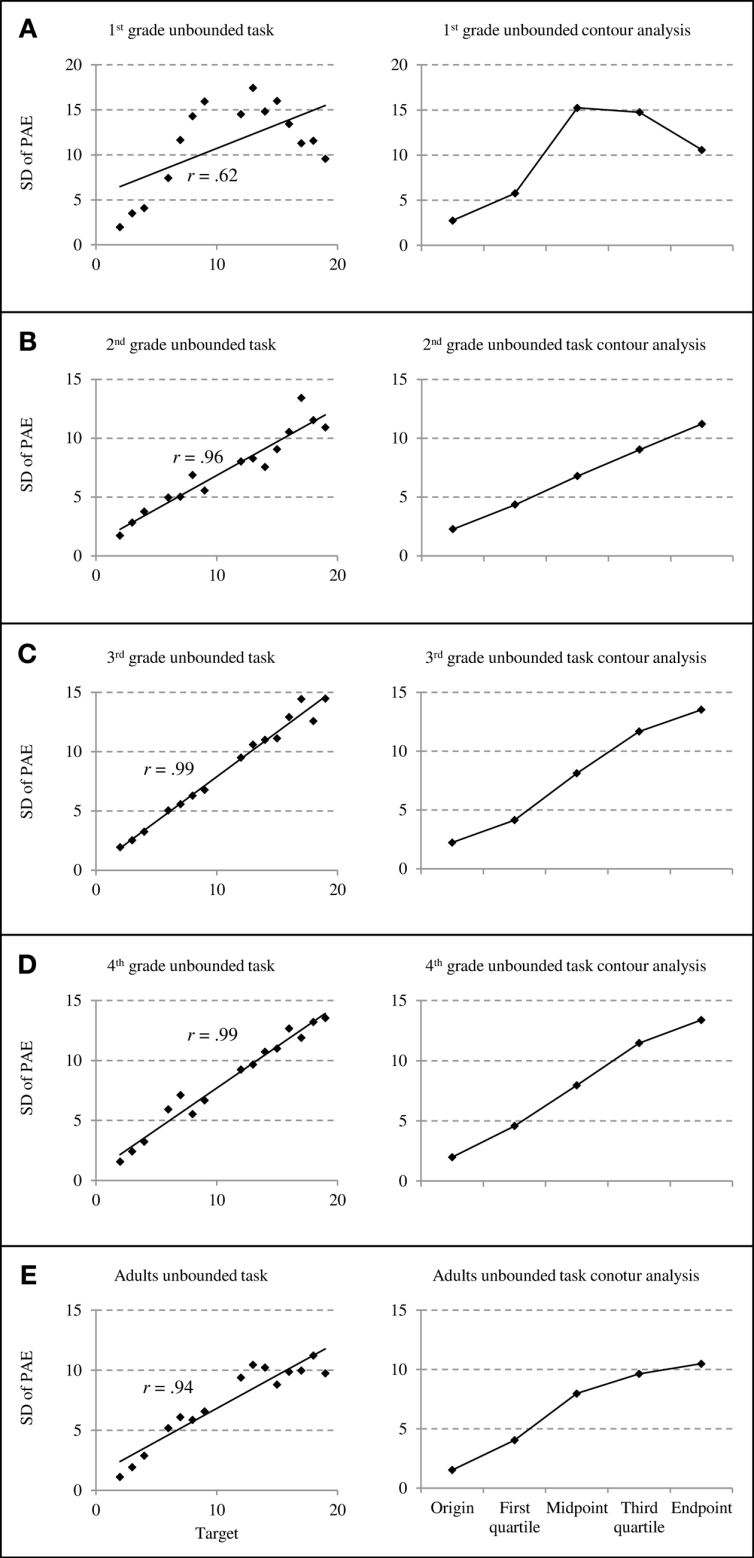
**Standard deviations of percent absolute errors (PAE) for unbounded number line estimation performance**. The left column depicts the relationship between standard deviations of PAEs and target numbers whereas the right column depicts results of counter analyses summarizing standard deviations of PAEs at specific reference points **(**cf. Ashcraft and Moore, 2012; grade 1 through adults: **A**–**E)**.

On the panels of Figures 3 and 4, we plotted the mean standard deviations of PAE as a function of target number (see left column) and the standard deviations of PAEs at targets close to specific reference points (i.e., origin, midpoint, and endpoint) and in between reference points (first quartile, third quartile) to be compared in a contour analysis (right column; cf. Ashcraft and Moore, 2012).

Even from visual inspection of bounded number line estimation performance, it is obvious that between grades two and three (Figures 3B,C) a change in children's estimation strategies seems to occur. First- and second-graders' PAE variability increased significantly with increasing target numbers as indicated by both, the more detailed distribution of PAE variability (Figures 3A,B, left column) as well as the contour analyses (Figures 3A,B, right column). Correlating SD of PAE and size of target number revealed significant correlations of *r* = 0.99 (*p* < 0.01) for both age groups. In contrast, children from grade three on showed *M*-shaped patterns of PAE distribution and no significant correlation between SD of PAE and size of target number (all *r* < 0.32, all *p* > 0.18). This means that children's estimations varied less at and around the to-be-expected reference points (i.e., start and endpoint as well as the midpoint of the scale) whereas PAE variability was reliably larger in between these reference points. Additionally, the same patterns were also present for fourth-graders and adults, especially when taking a closer look at the distribution of PAE variability plotted as target function (see Figures 3C–E, left column). Importantly, these (indicated) *M*-shaped patterns of error distribution are characteristic for proportion-judgment strategies (cf. Cohen and Blanc-Goldhammer, 2011). Generally, statistical evaluation by the contour analyses (see Figure 3, right column) substantiated these *M*-shaped patterns. The *t*-tests to evaluate whether PAE variability is indeed reduced at/around suspected reference points (start-, mid and endpoint) compared to in between them (first and third quartile) revealed no significant differences for first- and second-graders' PAE variability (both *t* < 0.88, both *p* > 0.40, one-sided) but indicated (marginally) significant smaller PAE variability at/around reference points for third-graders, adults (both *t* > 4.10, both *p* < 0.01, one-sided), and fourth-graders [*t*(8) = 2.19, *p* = 0.06, one-sided].

In contrast to this, for the unbounded number line task similar PAE distributions were observed across all age groups: PAE variability increased monotonously with target number (see Figures 4A–E) resulting in significant correlations between SD of PAE and size of target number for all age groups (from *r* = 0.62 to *r* = 0.99, all *p* < 0.05). This pattern of linearly increasing error variability was most prevalent for second-, third-, and fourth graders. However, for first-graders we observed PAE variability to decrease toward the end of the scale (see Figure 4A) while PAE variability remained constant for adults' estimates of larger target numbers (see Figure 4E). This pattern was due to the fact that first-graders and adults placed larger numbers toward the end of the physical line, thereby increasing PAEs but reducing their variability or holding it constant, respectively. Importantly, the *t*-tests statistically evaluating the contour analysis did not reveal any significant differences in PAE variability at/around reference points compared to PAE variability in between these reference points (all *t* < 0.50, all *p* > 0.63).

## Discussion

The current study set off to investigate the development of children's spatial representation of number magnitude by comparing their estimation performance in both a standard bounded as well as a new unbounded version of the number line estimation task. Such a direct contrast of the two versions of the task for children is of particular interest as a recent study with adult participants (Cohen and Blanc-Goldhammer, 2011) indicated that the standard, bounded number line estimation task seems to induce proportion-judgment strategies whereas the new, unbounded version of the task was supposed to provide a more pure measure of the underlying spatial magnitude representation. This was concluded by Cohen and Blanc-Goldhammer (2011) from the fact that no reduction of error variability at specific reference points was observed in unbounded number line estimation indicating that this task is better suited to make inferences on the representation of integer numbers along the mental number line. To investigate the development of possible differences between these two versions of the task we assessed children from first to fourth grade on the bounded as well as the unbounded number line estimation task in a cross-sectional design. In line with recent data (Schneider et al., 2008; Slusser et al., 2013) we expected to observe evidence for proportion-judgment strategies in children from grade three at the latest for the bounded but not the unbounded number line estimation task. The present data partially corroborated this hypothesis as we observed a qualitative change in estimation performance for bounded but not unbounded number line estimation with increasing age, in particular between second and third grade. Note, however, that Slusser et al. (2013) observed evidence for the predominant use of proportion-judgment strategies already in seven-year olds whereas in the current study this was only the case for third-graders and older children. Cultural or school characteristics or task attributes may explain this slight difference.

In the following, we will first discuss the differential development in the bounded and unbounded version of the number line estimation task before elaborating on the broader implication for research on children's numerical development.

### Differential development of number line estimations in bounded vs. unbounded number line tasks

First- and second-graders' bounded and unbounded number line estimations indicated no substantial differences between the estimation patterns and error distributions for the two versions of the number line estimation task in our study. This corroborates our hypothesis that indices for the use of proportion-judgment strategies (considering at least two reference points) may only occur after a certain level of proficiency has been reached (see also Slusser et al., 2013 for a similar argument, however, for an earlier start of proportion judgments). Importantly, this interpretation is corroborated by the results of the model fittings: For the bounded estimation task, we found the estimation pattern of most children to be fitted best by linear functions instead of cyclic models not indicating the use of proportion-judgment strategies. In line with this, unbounded power models were observed to fit best the estimates of most children in the unbounded number line estimation task—again not indicating the use of proportion-judgment strategies. Instead the prominently observed models indicate that children directly estimated target numbers without using reference points other than the start point (cf. Cohen and Blanc-Goldhammer, 2011).

Apart from this general pattern there was an interesting finding for the first-graders in our sample. We observed that first-graders overestimated positions of large numbers in the unbounded number line task. This means that they placed numbers close to 20 (which was the maximum of the number range assessed) even beyond than necessary (i.e., further to the right, see Figure 2, Panel A). Because we also observed a decrease of the variation of the estimation errors toward the end of the scale, this probably indicates that first-graders used the length of the physical line as any kind of orientation. However, because there was no numerical endpoint indicated and there is evidence that relatively younger children tend to even ignore the upper bound when given (at least they do not seem to use it systematically as a reference point, Slusser et al., 2013) we are confident that this does not suggest the use of proportion-judgment strategies. This is also backed by the modeling results with no indications for cyclic models to fit the data best. Rather, children seemed to consistently overestimate the target numbers with the largest numbers seeming so large to them, that they locate them toward the end of almost any unbounded number line. Synced with the fact that even first graders are usually able to adhere to the ordinal sequence of the numbers in number line estimations (e.g., Moeller et al., 2009) it is just a consequence of such behavior that error variation decreases toward the end of the physical line.

In contrast to first- and second-graders and in line with previous studies investigating bounded number line estimation (e.g., Slusser et al., 2013) estimation performance of relatively older children revealed explicit differences between the bounded and unbounded version of the number line task. For the unbounded number line estimation task estimation patterns as well as the monotonously increasing variation of estimation errors did not indicate the use of reference points. Again this was corroborated by the modeling results as we found the estimation patterns of the vast majority of children (third- and fourth-graders) to be fitted best by models indicating direct estimation strategies. However, inspection of both, estimation patterns as well as error variability indicated that this did not hold for estimation performance in the bounded number line estimation task. Although estimation patterns looked rather linear one- and two-cycle power models provided the best fit for the majority of children's' estimates - clearly indicating the use of either two (i.e., start and endpoint) or three reference points (i.e., start, middle and endpoint; cf. Cohen and Blanc-Goldhammer, 2011; Slusser et al., 2013). Furthermore, the variation of estimation errors showed the characteristic *M*-shaped distribution indicating that error variability decreased at these respective reference points (cf. Cohen and Blanc-Goldhammer, 2011). Thus, our results are in line with recent evidence suggesting that relatively older children probably from grade three on (Schneider et al., 2008, but see Slusser et al., 2013 for proportional judgment from grade two on), systematically rely on proportion-judgment strategies in number line estimation - but only so when performing the bounded number line task.

A similar pattern was observed when looking at adults' estimation performance. Although model fittings indicated adults to use direct estimation strategies a closer inspection of PAE variability was also informative. The *M*-shaped distribution of PAE variability clearly indicated the use of proportion-judgment strategies. Synced with the fact that model fittings can hardly differentiate between very accurate and basically linear estimation patterns the fitting results for adult participants should be considered only cautiously. In contrast, adults' estimates on the unbounded number line revealed no indication for the systematic use of proportion-judgment strategies. However, different from second- to fourth-graders and comparable to first-graders, adults seemed to use the end of the number line as some kind of endpoint. Not only did adults overestimate numbers close to the largest target number assessed but their PAE variability remained constant for these target numbers as well. This result pattern, however, is in line with previous findings of Cohen and Blanc-Goldhammer (2011) who removed participants' responses for the largest items from further analyses because “the computer screen boundary acted as an artificial endpoint and skewed these data low” (Cohen and Blanc-Goldhammer, 2011, p. 335). Importantly, even though adults may have tried to figure out the endpoint of the number line by locating the largest targets toward the end of the physical line, model fittings as well as visual inspection of PAE variability did not provide any evidence for the use of specific estimation strategies (i.e., prominent use of proportion-judgment or dead-reckoning strategy) in unbounded number line estimation.

Taken together and in line with previous studies (cf. Cohen and Blanc-Goldhammer, 2011; Slusser et al., 2013), our results suggest that the standard bounded number line estimation task seems to induce specific proportion-judgment strategies for relatively older children and adults. Therefore, these data add to recent evidence challenging the view that the bounded version of the number line estimation task allows for direct inferences about the nature of the spatial representation of number magnitude (see also Barth and Paladino, 2011; Karolis et al., 2011). Cohen and Blanc-Goldhammer (2011) proposed that not the estimation pattern but the error variability found in number line estimation tasks allows inferences about the representation of the magnitude of numbers. In line with their data for adults we found the error variability to increase linearly in the unbounded number line estimation task indicating a linear number representation with scalar variance (e.g., Gibbon and Church, 1981; Brannon et al., 2001). However, most importantly following the rationale of Cohen and Blanc-Goldhammer (2011) our data suggest that the unbounded version seems to provide a purer measure of number line estimation performance in children as well—at least for relatively older children while we were not able to find systematic differences between performance in bounded and unbounded number line estimation for the relatively younger participants in our study (first-and second-graders). Thus, for relatively younger children both versions of the task may tap on number line estimation whereas for older children performance in the bounded version may be complemented by strategies other than number line estimation. This is of particular interest from a developmental point of view because performance in the bounded version of the number line estimation task has repeatedly been associated with actual as well as future numerical achievement (e.g., Booth and Siegler, 2008).

### Implications for research on numerical development

Estimation performance in the bounded number line task is closely related to other numerical concepts, to some extent even causally (e.g., Booth and Siegler, 2008; Siegler and Ramani, 2009). Children with a more accurate linear representation are not only more proficient in other numerical tasks such as addition but are also better in learning new arithmetical problems. Yet, given the interpretation of Slusser et al. (2013), who suggested children to improve in number line estimation when able to consider more reference points to successfully apply proportion-judgment strategies, the question arises what it is that links performance in the bounded number line estimation task to other numerical concepts—acknowledging that it may not be (as originally proposed) the index of the underlying spatial magnitude representation? A possible account might consider the conceptual similarity of applying proportion-judgment strategies to some extent involve an understanding of part-whole relations and thus fractions and the concept of division (e.g., the midpoint requires an understanding of halving). Importantly, there is now accumulating evidence indicating that fraction understanding has a central role in numerical development as well as educational achievement, in particular beyond the first few years of schooling (see Siegler et al., 2013, for a review). Not only that high school students' fraction knowledge correlates very high with their actual mathematics achievement (*r* > 0.80). Fraction knowledge of relatively older children (i.e., fifth-graders) also predicts future algebra and overall mathematics achievement in high school even after taking into account covariates such as IQ, reading ability, working memory, etc. (Siegler et al., 2012, see also Bailey et al., 2012; Booth and Newton, 2012 for the influence of fraction understanding on mathematics achievement). Importantly, this is in accordance with educational and instructional practice. Lack of fraction knowledge was ranked to be amongst the most important problems hindering students' algebra learning by a representative sample of 1000 US algebra teachers (National Mathematics Advisory Panel, 2008a). And as a consequence, the National Mathematics Advisory Panel (2008b) asserts that “the teaching of fractions must be acknowledged as critically important and improved before an increase in student achievement in algebra can be expected” (p. 18). In this vein, we propose that for older children the concept of proportionality also or predominantly drives the observed predictive power of estimation performance in the bounded number line estimation task for actual and future numerical and arithmetical achievement. In sum, it may not be the spatial representation of number magnitude also assessed in the unbounded number line task, but rather proportional strategies specific to the bounded number line task which are related to other arithmetic competencies.

### Limitations and perspectives

Although it was not at the heart of the current study to compare the two versions of number line estimation tasks with respect to task difficulty, we wish to elaborate on potential concerns about the different number ranges assessed being responsible for the application of different strategies. The choice of different number ranges for the bounded task was based on the results of previous studies (cf. Slusser et al., 2013) which showed that already 8- to 10-year-olds are not only able to perform number line tasks in ranges up to 100,000 but also applied proportion-judgment strategies in these ranges. Therefore, the eventual concern that the bounded number line estimation task might have been more difficult for older children and adults simply because of the higher number ranges covered seems premature. This argument is further corroborated by a closer inspection of means and standard deviations of PAEs. As can be read from Table 2 estimation errors were higher for bounded number line performance than for unbounded number line performance only for first- and second-graders (both *t* > 3.1, both *p* < 0.01) although the assessed number ranges in the bounded task were either the same or even smaller compared to the range assessed in the unbounded number line estimation task. Interestingly, the reversed pattern was found for children older than grade two and adults: even though larger and supposedly more difficult number ranges were administered in the bounded task versions mean PAEs are higher for the unbounded task (all *t* > 5.8, all *p* < 0.01). This pattern nicely corroborates our hypothesis that older children and adults apply proportion-judgment strategies for solving bounded number line estimation (making these easier) whereas younger children are (not yet) able to apply such a strategy.

**Table 2:** **Mean PAE's (percent absolute error) and *SD* of PAEs for the range of the respective number line task separated for the different age groups**.

**Age group**	**Bounded task**			**Unbounded task**		
	***M***	***SD***	**Range**	***M***	***SD***	**Range**
First grade	22.8	11.6	0–10	15.0	7.6	0–20
Second grade	14.7	9.2	0–20	10.7	4.4	0–20
Third grade	6.4	2.8	0–100	13.8	6.6	0–20
Fourth grade	6.0	2.1	0–1000	11.9	6.1	0–20
Adults	3.3	1.1	0–10,000	10.0	5.7	0–20

An additional argument corroborating this interpretation comes from the development of PAEs. Comparing mean PAEs within bounded number line estimation, an ANOVA revealed a main effect of age group [*F*_(4, 239)_ = 68.75, *p* < 0.01]. *Post-hoc* pairwise comparisons showed significant differences between mean PAEs of first-graders and second-graders with all other age groups (all *p* < 0.01). Third- and fourth-grader's as well as adult's mean PAEs for the bounded task did not differ significantly. For mean PAEs for unbounded estimation an ANOVA revealed a main effect of age group [*F*_(4, 239)_ = 5.54, *p* < 0.01]. *Post-hoc* tests indicated significantly higher PAEs for first-graders compared to second-graders and adults (*p* < 0.05, *p* < 0.01, respectively). Additionally, third-graders were indexed to differ significantly from adults regarding their mean PAE (*p* < 0.05). So, constant PAEs for third-, fourth-graders and adults in the bounded number line task indicate that the difficulty of the respective range assessed was approximately the same for the different age groups. Since differences in estimation errors of unbounded number line performance reveal no systematic pattern (see Table 2, notification) there are no obvious indications for strategy application.

However, besides these issues regarding the ranges used in bounded number line estimation other theoretical as well as methodological aspects which we did not control for explicitly might also play a role in number line estimation performance. For example, differences in the visual appearance of the tasks, or the missing second landmark in the unbounded task, in particular, might involve different processes of spatial recall. For instance it is interesting to note that Huttenlocher et al. (1994; see also Hund and Spencer, 2003; Schutte et al., 2011), observed that children's performance pattern in spatial recall tasks were very similar to estimation patterns found for bounded number line estimation. When supposed to remember the spatial location of a hidden toy, children also relied on the boundaries of sandboxes as location cues. While younger children (4- to 7-year olds) showed a memory bias toward the middle of the sandbox older children (10- to 11-year olds) showed a bias toward the first and third quartile of the box (speaking in terms of number line segmentation; Huttenlocher et al., 1994). Thus, the application of proportion-judgment strategies in bounded number line estimation might also be influenced by more general aspects of spatial cognition. This assumption is further corroborated when considering the influence of spatial attention for number line performance. LeFevre et al. (2010; see also LeFevre et al., 2013) observed that besides linguistic and quantitative processes, spatial attention is a unique precursor for early numeracy skills, also predicting number line estimation performance.

In sum, this correspondence asks for further studies to disentangle how number line estimation is influenced by both general influences of spatial cognition and the particular spatial attributes of task presentation such as item placement or task instruction (cf. Barth and Paladino, 2011). It is well conceivable that such factors also influence estimation performance. For example, in our study, items of the bounded task were always depicted above the midpoint of the number line whereas items of the unbounded task were depicted above the start point. However, as our results obtained for adult participants are more or less identical to those of Cohen and Blanc-Goldhammer (2011) who controlled for item placement we would not assume children's estimation patterns to be distinct when item placement was controlled. In addition, there are other interesting and important research questions for future studies regarding the way performance in the unbounded number line estimation task relates to other actual but also future numerical and arithmetical competencies. Assuming unbounded number line estimation to provide a purer measure of the underlying spatial representation of number magnitude, evaluating its relationship with other numerical competencies might be of particular interest given the strong relationship observed for bounded number line estimation.

## Conclusions

In the current study we directly compared children's estimations in the standard bounded as well as a new unbounded version of the number line estimation task. In line with recent research we found reliable evidence for the use of proportion-judgment strategies in bounded number line estimation for relatively older children (third- and fourth-graders) and adults adding to evidence suggesting that estimations in the bounded number line task are not reflecting an isomorphic measure of the mental number line of relatively older children and adults.

In contrast, there were no indications for the use of any strategies other than direct number line estimation for the unbounded number line estimation task which, thus, may be a valuable tool for assessing the spatial magnitude representation in a more unbiased way, at least for older children and adults. The fact that we did observe similar results for the bounded and the unbounded version of the task for first- and second-graders may indicate that both versions of the task might assess the same underlying representation for relatively younger children at least in number ranges familiar to the children assessed.

Taken together, the bounded and the unbounded number line estimation task seem to assess different representations and processes, although aiming to assess the same underlying spatial representation of number magnitude. Importantly, this has implications on a broader level. As estimation performance in the bounded number line task is not only correlated with but even causally related to other numerical and arithmetic competencies, future research is necessary to investigate whether it is indeed the spatial representation of number magnitude (assessed by the bounded number line task) or rather the concomitantly assessed proportion understanding which predicts future numerical competencies and achievement.

### Conflict of interest statement

The authors declare that the research was conducted in the absence of any commercial or financial relationships that could be construed as a potential conflict of interest.

## Footnotes

^1^Please note that we previously advocated bi-linear or multi-linear fittings of children's number line estimations whereas we fitted one-cycle and two-cycle models (cf. Barth and Paladino, 2011; Slusser et al., 2013) in the current study. Importantly, we wish to emphasize that this is not a contradiction. In recent studies we fitted seemingly logarithmic estimation patterns using a bi-linear model and argued that what seems logarithmic actually reflects separate representations for single- and two-digit numbers possibly indicating insufficient place-value understanding (Moeller et al., 2009; Helmreich et al., 2011; Moeller and Nuerk, 2011). However, proper understanding of the base-10 place-value structure is actually a mandatory prerequisite for any proportional strategy because the proportions applied must be represented correctly (or at least roughly) to produce correct estimates. The idea that successful base-10 place-value understanding is inevitably necessary for the task and that proportional strategies are employed in later developmental stages are not incompatible with each other: The multi-linear account represents an alternative for seemingly logarithmic estimation patterns whereas the proportion-judgment account reflects an alternative account for seemingly linear (as compared to logarithmic) estimation patterns. Because children of all age groups consistently showed seemingly linear rather than logarithmic estimation patterns in the current study bilinear accounts, which suppose insufficient base-10 place-value understanding, are not applicable here.
